# As Mulheres estão Associadas a Menores Riscos de Mortalidade a Longo Prazo em Pacientes Submetidos à Revascularização do Miocárdio sem Circulação Extracorpórea

**DOI:** 10.36660/abc.20240221

**Published:** 2024-11-22

**Authors:** Chen Bai, Jiangang Wang, Qing Ye, Cheng Zhao

**Affiliations:** 1 Department of Cardiovascular Surgery Beijing Anzhen Hospital Capital Medical University Beijing China Department of Cardiovascular Surgery, Beijing Anzhen Hospital, Capital Medical University, Beijing – China

**Keywords:** Revascularização Miocárdica, Ponte de Artéria Coronária sem Circulação Extracorpórea, Mortalidade, Mulheres

## Abstract

**Fundamento:**

Em pacientes submetidos à cirurgia de revascularização do miocárdio sem circulação extracorpórea (CRM sem CEC), as mulheres apresentaram menores taxas de mortalidade hospitalar e fibrilação atrial pós-operatória do que homens. No entanto, a associação entre gênero e prognóstico de longo prazo após a CRM sem CEC raramente é relatada.

**Objetivo:**

Este estudo visa determinar se as mulheres se beneficiam mais da CRM sem CEC do que os homens, comparando a diferença no risco de mortalidade por todas as causas em longo prazo em pacientes com oclusão total crônica (CTO) coronária.

**Métodos:**

Este é um estudo de coorte observacional e retrospectivo conduzido em pacientes adultos (≥18 anos) com CTO coronária submetidos à CRM sem CEC em nosso hospital de janeiro de 2011 a dezembro de 2014. Um modelo multivariado de riscos proporcionais de Cox foi empregado para avaliar a associação de gênero com o risco de mortalidade por todas as causas. Um valor de p <0,05 foi considerado estatisticamente significativo.

**Resultados:**

Foram inscritos 1.256 participantes no total, dos quais 321 (25,6%) eram mulheres e 935 (74,4%) eram homens. Durante um acompanhamento máximo de 10 anos, a taxa de mortalidade por todas as causas em mulheres foi significativamente menor do que em homens (10,3% vs. 24,3%, p<0,01). A análise de regressão multivariada de Cox indicou que as mulheres estavam significativamente associadas a um menor risco de mortalidade por todas as causas (HR=0,30, IC 95%: 0,20-0,44; p<0,01) após o controle de potenciais fatores de confusão.

**Conclusão:**

As mulheres se beneficiam mais da cirurgia CRM sem CEC do que os homens, pois têm um menor risco de mortalidade por todas as causas em longo prazo em pacientes com CTO coronária.

## Graphical abstract

**Figura Central:**
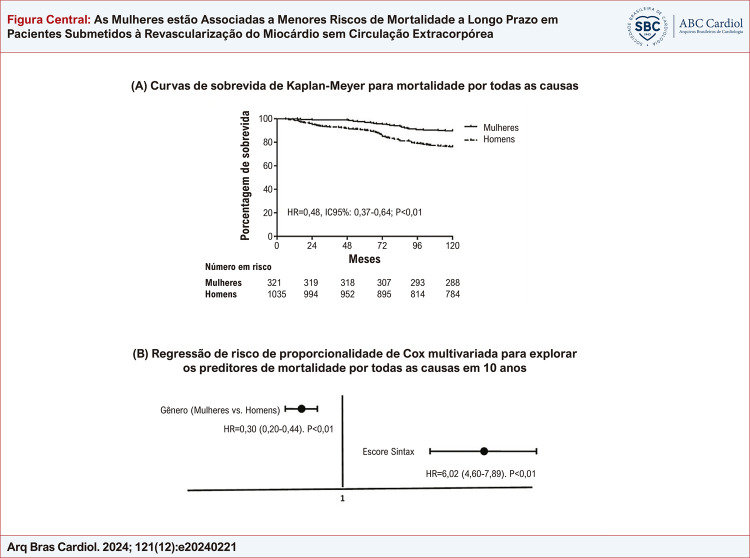
: As Mulheres estão Associadas a Menores Riscos de Mortalidade a Longo Prazo em Pacientes Submetidos à Revascularização do Miocárdio sem Circulação Extracorpórea

As mulheres estão associadas a menor risco de mortalidade a longo prazo em pacientes com oclusão total crônica passando por enxerto de revascularização da artéria coronária sem circulação extracorpórea.

## Introdução

A cirurgia de revascularização do miocárdio (CRM) é considerada a opção de tratamento mais popular para pacientes com doença arterial coronariana, podendo ser realizada de duas formas básicas: CRM com circulação extracorpórea (CEC) e CRM sem CEC.^1^ Devido ao aumento da experiência cirúrgica e anestésica e às melhorias técnicas nos dispositivos de estabilização intraoperatória do paciente, a aplicação da CRM sem CEC cresceu substancialmente,^2^ respondendo por mais de 80% de todas as CRM em países em desenvolvimento e no Extremo Oriente.^3,4^

Apesar de quase 2 décadas de debate com mais de 120 ensaios clínicos randomizados e 60 metanálises, a controvérsia sobre os benefícios e riscos de CRM sem CEC em comparação com CRM com CEC continua. No entanto, alguns estudos argumentam que CRM sem CEC é uma alternativa segura para CRM com CEC em pacientes do sexo feminino e é recomendado como uma técnica preferencial em mulheres. Uma metanálise de seis estudos observacionais envolvendo 23.313 pacientes (9.596 CRM sem CEC e 13.717 CRM com CEC) revelou um menor risco de infarto do miocárdio perioperatório em CRM sem CEC (OR=0,65; IC 95%: 0,51-0,84, p=0,0009).^5^ Em um estudo retrospectivo na Holanda, a mortalidade de 120 dias após CRM sem CEC foi significativamente menor do que após CRM com CEC em mulheres (OR=0,356, IC 95%: 0,144–0,882, p=0,026), e a diferença não foi significativa nos homens (OR= 0,787, IC 95%: 0,498–1,246, p=0,307).^6^ Mesmo em todos os pacientes submetidos à CRM sem CEC, as mulheres ainda apresentaram menores taxas de mortalidade hospitalar (0% vs. 1,3%) e fibrilação atrial pós-operatória (6% contra 15%) do que pacientes do sexo masculino.^7^ Entretanto, a associação de gênero e prognóstico a longo prazo após CRM sem CEC raramente é relatada.

O presente estudo tem como objetivo avaliar se as mulheres se beneficiam mais do que os homens da CRM sem CEC em pacientes com CTO crônica, que representa a lesão coronariana mais grave, com grande carga isquêmica e mau prognóstico.

## Métodos

### Pacientes

Este estudo de coorte observacional e retrospectivo incluiu pacientes adultos (≥18 anos) com CTO coronária submetidos a CRM sem CEC em nosso hospital de janeiro de 2011 a dezembro de 2014. CTO coronária foi definida como estenose de 100% com fluxo de grau 0 de trombólise no infarto do miocárdio (TIMI) por mais de 3 meses.^8^ Para minimizar a heterogeneidade da população, os pacientes foram excluídos se tivessem sido submetidos a refazer CRM. Todos os pacientes com morte perioperatória ou choque cardiogênico também foram excluídos.

Este estudo foi aprovado pelo Conselho de Revisão Institucional do Hospital Beijing Anzhen (2022178X). Todos os procedimentos foram conduzidos de acordo com a Declaração de Helsinque de 1964 e suas emendas posteriores ou padrões éticos comparáveis. O consentimento informado foi dispensado pelo Comitê de Ética do Hospital Beijing Anzhen devido à natureza retrospectiva do estudo. A participação do paciente foi anônima, e o nome ou informações de identificação não foram coletados.

### Operação CRM sem CEC

Todos os pacientes foram tratados com anestesia padrão, cirurgia e perioperatório por anestesistas, cirurgiões e enfermeiros experientes. Resumidamente, todos os pacientes foram levados para a sala de cirurgia e colocados na mesa em posição supina. Quando foram anestesiados, os cirurgiões fizeram uma incisão no meio do peito para separar o esterno e a veia safena magna; então, eles abriram o pericárdio e expuseram completamente os corações dos pacientes para explorar a estenose dos vasos sanguíneos. Os vasos apropriados para ponte foram escolhidos com base na localização da lesão vascular. Depois disso, os cirurgiões anastomosaram a aorta ascendente e a veia safena magna e suturaram o pericárdio.

### Coleta de dados e resultados

As características demográficas e clínicas basais dos pacientes foram coletadas de prontuários médicos, incluindo idade, sexo, índice de massa corporal (IMC), histórico de tabagismo, comorbidades (hipertensão, diabetes, doença arterial periférica e doença cerebrovascular), Score Synergy Between PCI With Taxus and Cardiac Surgery (Syntax), classe da New York Heart Association (NYHA), fração de ejeção e viabilidade miocárdica. Além disso, os dados perioperatórios, incluindo duração da operação, número de enxertos, tipo de enxertos (venoso, artéria mamária interna esquerda [AMIE], artéria mamária interna direita [AMID] e radial), duração pós-operatória de internações na unidade de terapia intensiva (UTI), duração pós-operatória de internações hospitalares e medicamentos na alta também foram recuperados. O desfecho primário foi a mortalidade por todas as causas. As informações sobre a morte foram coletadas por meio da revisão de registros de pacientes ou por entrevista por telefone. O acompanhamento terminou dez anos após a cirurgia ou em dezembro de 2020, o que ocorresse primeiro.

### Análise estatística

Estimamos que uma amostra de pelo menos 300 pacientes por grupo forneceria >90% de poder para detectar uma redução relativa de 10% no risco de morte no grupo feminino, em comparação com o grupo masculino, em um nível alfa bilateral de 0,05. O risco relativo estimado foi baseado em resultados de estudos anteriores que exploraram as diferenças nas taxas de mortalidade entre os gêneros.^9,10^

Variáveis contínuas com distribuição normal foram relatadas como média ± desvio padrão (DP) e comparadas usando testes-t de Student não pareados. Variáveis com distribuição não normal foram apresentadas como mediana (interquartil intervalo) e comparadas usando o teste U de Mann-Whitney. As variáveis categóricas foram resumidas como contagens e porcentagens, e as comparações foram feitas usando o teste qui-quadrado ou o teste exato de Fisher, conforme apropriado. Um teste de log-rank e uma curva de Kaplan-Meier foram empregados para avaliar as diferenças no tempo até a mortalidade por todas as causas entre homens e mulheres. Um modelo de risco proporcional multivariado de Cox foi utilizado para avaliar a associação de gênero e mortalidade por todas as causas após o ajuste para potenciais fatores de confusão. Um valor de p < 0,05 foi definido como estatisticamente significativo. O pacote de programa de software estatístico SPSS (versão 22.0) foi usado para análise de dados.

## Resultados

Foram inscritos 1.356 pacientes no total, dos quais 321 (23,7%) eram mulheres e 1.035 (76,3%) homens. As variáveis demográficas e clínicas basais são descritas na Tabela 1. As mulheres eram 4,7 anos mais velhas no início e tinham um IMC significativamente menor do que os homens (ambos p<0,01). As incidências de hipertensão e doença cerebrovascular foram substancialmente maiores em mulheres, enquanto a incidência de tabagismo foi significativamente maior em homens (todos p<0,01). As mulheres tiveram uma proporção significativamente maior de escore Syntax>32 do que os homens (p=0,01). A Tabela 2 exibe as características perioperatórias entre homens e mulheres. A duração operatória foi significativamente maior em homens do que em mulheres (p<0,01). Não houve diferenças significativas no número de enxertos, tipo de enxertos, duração pós-operatória de internações na UTI, duração pós-operatória de internações hospitalares e medicamentos na alta. Durante um período máximo de acompanhamento de 10 anos, a mortalidade cumulativa por todas as causas em pacientes do sexo feminino foi significativamente menor do que em pacientes do sexo masculino (10,3% vs. 24,3%, p<0,01). A curva de sobrevida de Kaplan-Meier (Ilustração Central) revelou sobrevida cumulativa superior em pacientes do sexo feminino (HR=0,48, IC 95%: 0,37-0,64; p<0,01). A análise de regressão de riscos proporcionais de Cox multivariada foi empregada para avaliar a associação do gênero com o risco de mortalidade por todas as causas (Ilustração Central). Mostrou que após controlar potenciais fatores de confusão, incluindo características demográficas e clínicas basais dos pacientes (idade, sexo, IMC, histórico de tabagismo, comorbidades, escore Syntax, classe NYHA, fração de ejeção e viabilidade miocárdica) e dados perioperatórios (duração da operação, número de enxertos, tipo de enxertos, duração pós-operatória de internações na UTI, duração pós-operatória de internações hospitalares e medicamentos na alta), pacientes do sexo feminino foram significativamente associadas a um menor risco de mortalidade por todas as causas em comparação com pacientes do sexo masculino (HR = 0,30, IC de 95%: 0,20-0,44; p < 0,01), e pacientes com um escore Syntax mais alto foram significativamente associados a um maior risco de mortalidade por todas as causas (HR = 6,02, IC de 95%: 4,60-7,89; p < 0,01).

**Tabela 1:** – Características demográficas e clínicas basais

	Mulheres (n=321)	Homens (n=1.035)	p-valor
Idade, anos	68,0±3,0	63,3±3,0	<0,01
**IMC, kg/m^2^**	22,3±1,9	23,0±2,0	<0,01
Histórico de tabagismo	89 (27,7%)	595 (57,5%)	<0,01
Hipertensão	213 (66,4%)	479 (46,3%)	<0,01
Diabetes	79 (24,6%)	270 (26,1%)	0,61
Doença arterial periférica	36 (11,2%)	155 (15,0%)	0,10
Doença cerebrovascular	35 (10,9%)	59 (5,7%)	<0,01
Escore de sintaxe >32	142(44,2%)	376 (36,3%)	0,01
Classe NYHA			0,70
Eu-II	181 (56,4%)	569 (55,0%)	
III-IV	140 (43,6%)	466 (45,0%)	
**Fração de ejeção≥55%**	244 (76,0%)	801 (77,4%)	0,60
Falta de viabilidade miocárdica	20 (6,2%)	46 (4,4%)	0,23

IMC: índice de massa corporal; Sintaxe: Sinergia entre Intervenção Coronária Percutânea com Taxus e Cirurgia Cardíaca; NYHA: Associação Cardíaca de Nova York.

**Tabela 2:** – Dados perioperatórios

	Mulheres (n=321)	Homens (n=1.035)	p-valor
Duração operatória, min	158,7±5,2	159,7±4,9	<0,01
Número de enxertos	2,1±1,0	2,1±0,9	0,47
**Enxertos venosos**			
0	96 (29,9%)	317 (30,6%)	0,95
1	176 (54,8%)	566 (54,7%)	
2	201 (14,8%)	152 (14,7%)	
**AMIE**			0,65
0	60 (18,7%)	182 (17,6%)	
1	261 (81,3%)	853 (82,4%)	
**AMID**			0,08
0	287 (89,4%)	957 (92,5%)	
1	34 (10,6%)	78 (7,5%)	
**Renxertos adialis**			0,70
0	205 (63,9%)	673 (65,0%)	
1	116 (36,1%)	362 (35,0%)	
Duração pós-operatória de internação na UTI, dias	2,5±0,8	2,4±0,8	0,06
Duração da internação hospitalar pós-operatória, dias	6,8±1,8	6,9±1,7	0,51
**Medicamentos na alta**			
Estatinas	74 (23,1%)	287 (27,7%)	0,10
Aspirina	278 (86,6%)	919 (88,8%)	0,29
Tienopiridinas	56 (17,4%)	199 (19,2%)	0,48
IECA	45 (14,0%)	155 (15,0%)	0,67
BRA	45 (14,0%)	160 (15,5%)	0,53
antagonista β	35 (10,9%)	118 (11,4%)	0,84
Antagonista do cálcio	201 (62,6%)	653 (63,1%)	0,88
Nitratos	160 (49,8%)	475 (45,9%)	0,22

AMIE: artéria mamária interna esquerda; AMID: artéria mamária interna direita; IECA: inibidores da enzima de conversão da angiotensina; BRA: bloqueadores dos receptores da angiotensina.

## Discussão

Neste estudo, descobriu-se que as mulheres tinham uma menor taxa de mortalidade por todas as causas. Além disso, a regressão multivariada de Cox indicou que pacientes mulheres estavam significativamente associadas a um risco menor de mortalidade por todas as causas após o controle de potenciais fatores de confusão.

Conforme demonstrado em nossos resultados e em muitos estudos anteriores,^11,12^ as mulheres eram geralmente mais velhas, com mais comorbidades e maiores escores Syntax do que os homens no início do estudo. Um número crescente de estudos relatou que as mulheres tiveram mortalidade hospitalar ou de curto prazo significativamente maior do que os homens submetidos à CRM. As mulheres também foram consideradas um fator de risco em escores prevalentes para avaliar riscos perioperatórios, como o Sistema Europeu de Avaliação de Risco Operatório Cardíaco (EuroSCORE). Ao contrário da mortalidade de curto prazo, nosso estudo indicou uma incidência significativamente menor de mortalidade por todas as causas a longo prazo em mulheres. Há resultados conflitantes em estudos anteriores sobre a diferença de sexo a longo prazo na mortalidade após CRM sem CEC. Em linha com nossos resultados, um estudo publicado recentemente na Finlândia^13^ mostrou que a taxa de mortalidade por todas as causas em mulheres durante um acompanhamento de 10 anos foi 0,70 vezes (IC de 95%, 0,58–0,84; p < 0,0001) do que em homens. No entanto, estudos com 8-11 anos de acompanhamento do Canadá^9^ e Noruega^14^ não encontraram diferenças de gênero no prognóstico. Os resultados conflitantes entre os estudos podem ser devidos a diferentes populações, desenhos de estudo, fatores de confusão e eras de estudo.

A diferença de gênero na sobrevivência a longo prazo após CRM sem CEC pode estar relacionada a fatores de risco autoadquiridos, incluindo estado hormonal e adesão a medicamentos e estilos de vida saudáveis. Foi relatado que o estrogênio pode proteger altamente o sistema cardiovascular de mulheres na pós-menopausa e melhorar sua sobrevivência.^15^ Além disso, as mulheres tiveram melhor adesão à medicação e melhores estilos de vida, como menor consumo de álcool e menor taxa de tabagismo,^16,17^o que pode estar associado a melhor sobrevivência. Os mecanismos subjacentes para diferenças de gênero no prognóstico devem ser complicados e precisam ser explorados em estudos posteriores. Existem várias limitações, primeiro, devido ao desenho retrospectivo, não podemos excluir o viés de seleção e uma possível falta de variáveis que poderiam ter influenciado os resultados. Por exemplo, a adesão à medicação e o estilo de vida saudável (por exemplo, exercícios e cessação do tabagismo) durante o acompanhamento de longo prazo estavam ausentes, o que pode afetar a sobrevivência e causar viés. Além disso, as medidas de desfecho primárias foram mortalidade por todas as causas, e não recuperamos dados sobre a causa da morte.

## Conclusão

Em conclusão, o gênero feminino é um fator de risco independente para aumento das taxas de mortalidade pós-operatória quando CRM é usada. A CRM sem CEC está significativamente associada com melhor sobrevida a longo prazo em mulheres com CTO coronária e pode, portanto, ser proposta como a técnica de revascularização preferida entre pacientes do sexo feminino.
