# Supplementary material for: As Mulheres estão Associadas a Menores Riscos de Mortalidade a Longo Prazo em Pacientes Submetidos à Revascularização do Miocárdio sem Circulação Extracorpórea
Source: Arq Bras Cardiol. 2024 Nov 22;121(12):e20240221. [Article in Portuguese] doi: 10.36660/abc.20240221 (PMC11634319; doi:10.36660/abc.20240221)
Supplement: Translation

# Translation: Females are Associated with Lower Risks of Long-Term Mortality in Patients Undergoing Off-Pump Coronary Artery Bypass Grafting

**DOI:** 10.36660/abc.20240221i

**Authors:** Chen Bai, Jiangang Wang, Qing Ye, Cheng Zhao

**Affiliations:** 1 China Department of Cardiovascular Surgery, Beijing Anzhen Hospital, Capital Medical University, Beijing – China

**Keywords:** Myocardial Revascularization, Beating Heart Coronary Artery Bypass, Mortality, Women

## Abstract

**Background:**

In patients who underwent off-pump coronary artery bypass grafting (OPCABG), females were reported to have lower rates of in-hospital mortality rate and postoperative atrial fibrillation than males. However, the association between gender and long-term prognosis following OPCABG is rarely reported.

**Objective:**

This study aims to determine if women benefit more from OPCABG than men by comparing the difference in the risk of long-term all-cause mortality in patients with coronary chronic total occlusion (CTO).

**Methods:**

This is an observational, retrospective cohort study conducted in adult patients (≥18 years) with coronary CTO undergoing OPCABG at our hospital from January 2011 to December 2014. A multivariate Cox proportional hazards model was employed to assess the association of gender with the risk of all-cause mortality. A p-value of <0.05 was considered statistically significant.

**Results:**

Totally 1,256 participants were enrolled, among which 321 (25.6%) were females and 935 (74.4%) were males. During a maximum follow-up of 10 years, the all-cause mortality rate in women was significantly lower than that in men (10.3% vs. 24.3%, p<0.01). Multivariate Cox regression analysis indicated that women were significantly associated with a lower risk of all-cause mortality (HR=0.30, 95% CI: 0.20-0.44; p<0.01) after controlling for potential confounding factors.

**Conclusion:**

Women benefit more from OPCABG surgery than men, as they have a lower risk of long-term all-cause mortality in patients with coronary CTO.

## Introduction

Coronary artery bypass grafting (CABG) is regarded as the most popular treatment option for patients with coronary artery disease (CAD), which can be performed in two basic ways: on-pump CABG (ONCABG) and off-pump CABG (OPCABG).^1^ Owing to increases in surgical and anesthesia expertise and technical improvements in intraoperative patient stabilization devices, the application of OPCABG has grown substantially,^2^ accounting for over 80% of all CABG in developing countries and the Far East.^3,4^

Despite nearly 2 decades of debate with more than 120 randomized clinical trials (RCTs) and 60 meta-analyses, the controversy regarding the benefits and risks of OPCABG compared with ONCABG continues. However, some studies argue that OPCAB is a safe alternative to ONCABG in female patients and is recommended as a preferred technique in females. A meta-analysis of six observational studies involving 23,313 patients (9,596 OPCAB and 13,717 ONCABG) revealed a lower risk of peri-operative myocardial infarction in OPCABG (OR=0.65; 95% CI: 0.51- 0.84, p=0.0009).^5^ In a retrospective study in the Netherlands, the 120-day mortality after OPCABG was significantly lower than that after ONCABG in women (OR=0.356, 95% CI: 0.144–0.882, p=0.026), and the difference was not significant in men (OR= 0.787, 95% CI 0.498–1.246, p=0.307).^6^ Even in all patients who underwent OPCABG, females still had lower rates of in-hospital mortality rate (0% vs. 1.3%) and postoperative atrial fibrillation (6% vs. 15%) than male patients.^7^ However, the association of gender and long-term prognosis following OPCABG is rarely reported.

The present study aims to evaluate if women benefit more than men from OPCABG in patients with coronary chronic total occlusion (CTO), which represents the most severe coronary lesion with heavy ischemic burden and poor prognosis.

## Methods

### Patients

This observational, retrospective cohort study enrolled adult patients (≥18 years) with coronary CTO undergoing OPCABG at our hospital from January 2011 to December 2014. Coronary CTO was defined as a 100% stenosis with thrombolysis in myocardial infarction (TIMI) grade 0 flow for more than 3 months.^8^ To minimize the heterogeneity of the population, patients were excluded if they underwent re-do CABG. All patients with peri-operative death or cardiogenic shock were also excluded.

This study was approved by the Institutional Review Board of Beijing Anzhen Hospital (2022178X). All procedures were conducted according to the 1964 Declaration of Helsinki and its later amendments or comparable ethical standards. Informed consent was waived by the Ethic Committee of Beijing Anzhen Hospital because of the retrospective nature of the study. Patient participation was anonymous, and the name or identifying information was not collected.

### OPCABG operation

All patients were treated with standard anesthesia, surgery, and peri-operative nurses from experienced anesthetists, surgeons, and nurses. Briefly, all patients were brought to the operating room and placed on the table in the supine position. When they were anesthetized, surgeons made an incision down the middle of the chest to separate the sternum and the great saphenous vein; then, they opened the pericardium and fully exposed patients’ hearts to explore the stenosis of blood vessels. The appropriate bridging vessels were chosen based on the location of the vascular lesion. After that, surgeons anastomosed the ascending aorta and the great saphenous vein and sutured the pericardium.

### Data collection and outcomes

Patients’ baseline demographic and clinical features were collected from medical records, including age, gender, body mass index (BMI), smoking history, comorbidities (hypertension, diabetes, peripheral artery disease, and cerebrovascular disease), Synergy Between PCI With Taxus and Cardiac Surgery (Syntax) score, New York Heart Association (NYHA) class, ejection fraction, and myocardial viability. In addition, the peri-operative data, including operation duration, number of grafts, type of grafts (venous, left internal mammary artery [LIMA], right internal mammary artery [RIMA], and radialis), postoperative length of intensive care unit (ICU) stays, postoperative length of hospital stays, and medications at discharge were also retrieved. The primary endpoint was all-cause mortality. Death information was collected by reviewing patient records or by phone interview. The follow-up ended ten years after the surgery or in December 2020, whichever came first.

### Statistical analysis

We estimated that a sample of at least 300 patients per group would provide >90% power to detect a relative reduction of 10% in the risk of death in the female group, as compared with the male group, at a two-sided alpha level of 0.05. The estimated relative risk was based on results from earlier studies that explored the differences in mortality rates between genders.^9,10^

Continuous variables with a normal distribution were reported as mean ± standard deviation (SD) and compared using unpaired Student’s t-tests. Variables with a non-normal distribution were presented as median (interquartile range) and compared using the Mann-Whitney U test. Categorical variables were summarized as counts and percentages, and comparisons were made using the chi-square test or Fisher’s exact test as appropriate. A log-rank test and Kaplan-Meier curve were employed to evaluate differences in time to all-cause mortality between males and females. A Multivariate Cox proportional hazard model was utilized to assess the association of gender and all-cause mortality after adjusting for potential confounders. A p-value of <0.05 was set to be statistically significant. The SPSS statistical software program package (version 22.0) was used for data analysis.

## Results

Totally 1,356 patients were enrolled, among which 321 (23.7%) were women and 1,035 (76.3%) were men. Baseline demographic and clinical variables are described in Table 1. Women were older by 4.7 years at baseline and had a significantly lower BMI than men (both p<0.01). The incidences of hypertension and cerebrovascular disease were substantially higher in women, while the incidence of smoking was significantly higher in men (all p<0.01). Women had a significantly higher proportion of Syntax score>32 than men (p=0.01).

**Table 1:** – Baseline demographic and clinical characteristics

	Females (n=321)	Males (n=1,035)	p-value
Age, years	68.0±3.0	63.3±3.0	<0.01
**BMI, kg/m^2^**	22.3±1.9	23.0±2.0	<0.01
Smoking history	89 (27.7%)	595 (57.5%)	<0.01
Hypertension	213 (66.4%)	479 (46.3%)	<0.01
Diabetes	79 (24.6%)	270 (26.1%)	0.61
Peripheral artery disease	36 (11.2%)	155 (15.0%)	0.10
Cerebrovascular disease	35 (10.9%)	59 (5.7%)	<0.01
Syntax score >32	142(44.2%)	376 (36.3%)	0.01
NYHA class			0.70
I-II	181 (56.4%)	569 (55.0%)	
III-IV	140 (43.6%)	466 (45.0%)	
**Ejection fraction≥55%**	244 (76.0%)	801 (77.4%)	0.60
Lack of myocardial viability	20 (6.2%)	46 (4.4%)	0.23

IBMI: body mass index; Syntax: synergy between percutaneous coronary intervention with taxus and cardiac surgery; NYHA: New York Heart Association.

Table 2 displays the peri-operative features between men and women. Operative duration was significantly longer in males than in females (p<0.01). There were no significant differences in the number of grafts, type of grafts, postoperative length of ICU stays, postoperative length of hospital stays, and medications at discharge.

**Table 2:** – Perioperative data

	Females (n=321)	Males (n=1,035)	p-value
Operative duration, min	158.7±5.2	159.7±4.9	<0.01
Number of grafts	2.1±1.0	2.1±0.9	0.47
**Venous grafts**			
0	96 (29.9%)	317 (30.6%)	0.95
1	176 (54.8%)	566 (54.7%)	
2	201 (14.8%)	152 (14.7%)	
**LIMA**			0.65
0	60 (18.7%)	182 (17.6%)	
1	261 (81.3%)	853 (82.4%)	
**RIMA**			0.08
0	287 (89.4%)	957 (92.5%)	
1	34 (10.6%)	78 (7.5%)	
**Radialis grafts**			0.70
0	205 (63.9%)	673 (65.0%)	
1	116 (36.1%)	362 (35.0%)	
Postoperative length of ICU stays, days	2.5±0.8	2.4±0.8	0.06
Postoperative length of hospital stays, days	6.8±1.8	6.9±1.7	0.51
**Medications at discharge**			
Statins	74 (23.1%)	287 (27.7%)	0.10
Aspirin	278 (86.6%)	919 (88.8%)	0.29
Thienopyridines	56 (17.4%)	199 (19.2%)	0.48
ACEI	45 (14.0%)	155 (15.0%)	0.67
ARB	45 (14.0%)	160 (15.5%)	0.53
β antagonist	35 (10.9%)	118 (11.4%)	0.84
Calcium antagonist	201 (62.6%)	653 (63.1%)	0.88
Nitrates	160 (49.8%)	475 (45.9%)	0.22

LIMA: left internal mammary artery; RIMA: right internal mammary artery; ACE: angiotensin-converting enzyme inhibitors; ARB: angiotensin receptor blockers.

During a maximum follow-up period of 10 years, the cumulative all-cause mortality in female patients was significantly lower than that in male patients (10.3% vs. 24.3%, p<0.01). The Kaplan-Meier survival curve (Central Illustration) revealed superior cumulative survival in female patients (HR=0.48, 95% CI: 0.37-0.64; p<0.01).

Multivariate Cox proportional hazards regression analysis was employed to evaluate the association of gender with the risk of all-cause mortality (Central Illustration). It showed that after controlling for potential confounders including patients’ baseline demographic and clinical features (age, gender, BMI, smoking history, comorbidities, Syntax score, NYHA class, ejection fraction, and myocardial viability) and peri-operative data (operation duration, number of grafts, type of grafts, postoperative length of ICU stays, postoperative length of hospital stays, and medications at discharge), female patients were significantly associated with a lower risk of all-cause mortality compared to male patients (HR=0.30, 95 %CI: 0.20-0.44; p<0.01), and patients with a higher syntax score were significantly associated with a higher risk of all-cause mortality (HR=6.02, 95% CI: 4.60-7.89; p<0.01).

## Discussion

In this study, females were found to have a lower all-cause mortality rate. Moreover, multivariate Cox regression indicated that female patients were significantly associated with a lower risk of all-cause mortality after controlling for potential confounders.

As shown in our results and many previous studies,^11,12^ women were generally older with more comorbidities and higher Syntax scores than men at baseline. An increasing number of studies have reported that females had significantly higher in-hospital or short-term mortality than males who underwent CABG. Females have also been regarded as a risk factor in prevalent scores for assessing peri-operative risks, such as the European System for Cardiac Operative Risk Evaluation (EuroSCORE). Contrary to short-term mortality, our study indicated a significantly lower incidence of long-term all-cause mortality in females. There are conflicting results in previous studies on the long-term sex difference in mortality after OPCABG. In line with our outcomes, a recently published study in Finland^13^ showed that the all-cause death rate in females during a 10-year follow-up was 0.70 times (95% CI, 0.58–0.84; p<0.0001) than that in males. However, studies with 8-11 years of follow-up from Canada^9^ and Norway^14^ did not find gender differences in the prognosis. The conflicting outcomes across studies may be due to different populations, study designs, confounding factors, and study eras.

The gender difference in long-term survival following OPCABG may be related to self-acquired risk factors, including hormonal status and adherence to medicines and healthy lifestyles. It has been reported that estrogen could highly protect the cardiovascular system of postmenopausal females and improve their survival.^15^ Moreover, females had better medication adherence and better lifestyles, such as lower alcohol consumption and a lower smoking rate,^16,17^ which might be associated with better survival. The underlying mechanisms for gender differences in prognosis should be complicated and need to be explored in further studies.

There are several limitations. Firstly, because of the retrospective design, we cannot exclude selection bias and a possible lack of variables that could have influenced the results. For instance, medication adherence and healthy lifestyle (e.g., exercise and smoking cessation) during the long-term follow-up were lacking, which may affect survival and cause bias. In addition, the primary outcome measures were all-cause mortality, and we did not retrieve data on the cause of death.

## Conclusion

In conclusion, the female gender is an independent risk factor for increased postoperative mortality rates when CABG is used. OPCABG is significantly associated with better long-term survival in women with coronary CTO and may, therefore, be proposed as the preferred revascularization technique among female patients.
